# Developing a real-time social distancing detection system based on YOLOv4-tiny and bird-eye view for COVID-19

**DOI:** 10.1007/s11554-022-01203-5

**Published:** 2022-02-22

**Authors:** Sergio Saponara, Abdussalam Elhanashi, Qinghe Zheng

**Affiliations:** 1 Dip. Ingegneria Informazione, University of Pisa, Via G. Caruso 16, 56122 Pisa, Italy; 2 School of Information Science and Engineering, Shandong University, Jinan, China

**Keywords:** Real-time video detection, COVID-19, Social distancing, YOLOv4-tiny, Bird’ eye view, Body temperature, Nvidia Jetson devices

## Abstract

COVID-19 is a virus, which is transmitted through small droplets during speech, sneezing, coughing, and mostly by inhalation between individuals in close contact. The pandemic is still ongoing and causes people to have an acute respiratory infection which has resulted in many deaths. The risks of COVID-19 spread can be eliminated by avoiding physical contact among people. This research proposes real-time AI platform for people detection, and social distancing classification of individuals based on thermal camera. YOLOv4-tiny is proposed in this research for object detection. It is a simple neural network architecture, which makes it suitable for low-cost embedded devices. The proposed model is a better option compared to other approaches for real-time detection. An algorithm is also implemented to monitor social distancing using a bird’s-eye perspective. The proposed approach is applied to videos acquired through thermal cameras for people detection, social distancing classification, and at the same time measuring the skin temperature for the individuals. To tune up the proposed model for individual detection, the training stage is carried out by thermal images with various indoor and outdoor environments. The final prototype algorithm has been deployed in a low-cost Nvidia Jetson devices (Xavier and Jetson Nano) which are composed of fixed camera. The proposed approach is suitable for a surveillance system within sustainable smart cities for people detection, social distancing classification, and body temperature measurement. This will help the authorities to visualize the fulfillment of the individuals with social distancing and simultaneously monitoring their skin temperature.

## Introduction

COVID-19 [1] is an acute respiratory infectious disease caused by a new coronavirus infection. It is mainly manifested by fever, dry cough, fatigue, etc. A small number of patients are accompanied by nasal congestion, runny nose, diarrhea and other upper respiratory and digestive tract symptoms. Severe cases often have difficulty breathing after 1 week, and severe cases rapidly progress to uncorrectable metabolic acidosis, coagulation dysfunction, and multiple organ failure. Until now, COVID-19 has caused more than 3 million deaths in many countries around the world. At present, many regions have adopted measures such as restricting traffic travel and canceling large-scale gatherings to prevent the spread of the virus. However, the new crown pneumonia epidemic has developed with the characteristics of a pandemic. In the next step, how to avoid the spread of the virus as much as possible in a normal environment is an urgent problem to be solved.

**Fig. 1:**
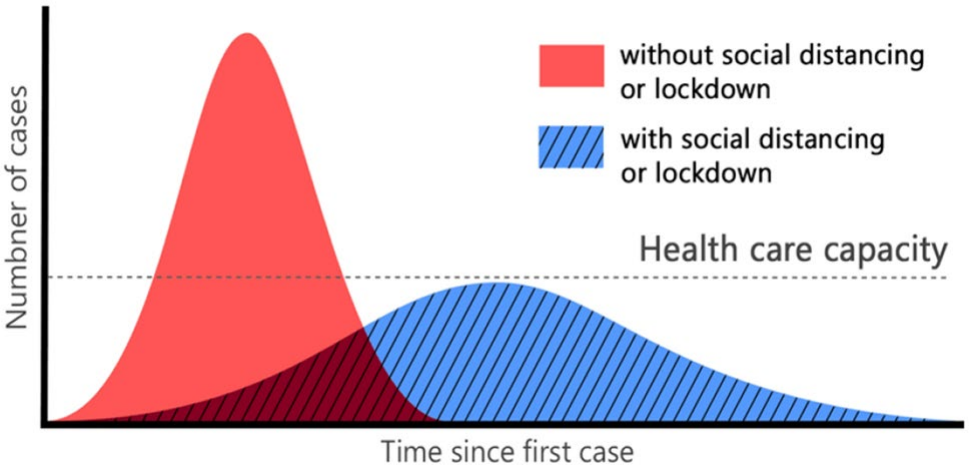
Gaussian curve that illustrates the distribution virus spread rate among the individuals, with and without applying the social distancing

Gaussian curves illustrate a little spike within the effectiveness of the health system, which makes it an easy approach for patients to prevent the virus by admiring persistent advice from health care authorities. Any unforeseen sharpened spike and rapid increase of the infection rate (such as the red curve in Fig. 1, will cause a health care service failure, and thus, growth in the number of deaths. Figure 1 illustrates the importance of following the guidelines of applying social distancing to minimize the spread of the virus among individuals [2, 3]

Although some vaccines [4] have been developed to suppress the spread of the virus, the most effective way is to maintain a safe social distance between pedestrians. Social distance refers to staying away from mass gatherings and keeping a distance of 6 feet from each individual—about the length of a body. Social distance is different from isolation and quarantine. Social distance is a preventive measure taken by the government for all. For those who have been infected or are believed to have been infected with infectious diseases, they need to be isolated in the ward and cared for by special medical personnel. Quarantine is for people who have been exposed to infected people but have not yet developed the disease. This means that effective pedestrian detection and distance measurement methods can help curb the spread of COVID-19. The most used pedestrian detection method is based on a computer vision solution in a public scene [5]. With the help of existing public area security cameras, pedestrian detection and social distance measurement can be realized conveniently and at low cost. Compared with the schemes based on mobile devices such as GPS sensors, the pedestrian detection methods based on computer vision have a wider range of applications, including intelligent-assisted driving [6], intelligent monitoring [7], pedestrian analysis [8], and intelligent robot [9]. Moreover, there are many open-source data sets of pedestrian detection based on computer vision that have been established to help evaluate and improve detection algorithms, such as the INRIA person dataset [10], Caltech pedestrian detection benchmark [11], and ETH dataset [12].

In the past, background modeling methods [13] were often used to extract foreground moving targets, and then feature extraction was performed in the target area and classifiers (e.g., multi-layer perceptron, support vector machine, and random forest) were used to classify them to determine whether pedestrians are included. In fact, it still faces the following problems in the actual application process: (1) changes in illumination can easily cause dramatic changes in image gray levels, thereby affecting detection accuracy. (2) Camera shake can easily cause the failure of background modeling, which affects the calculation of the target position. (3) There may be ghost areas that affect the judgment of the model. The method based on statistical learning [14] automatically mines features based on a large number of samples and constructs a pedestrian detection classifier. The extracted features mainly include the grayscale, edge, texture, color, and gradient histogram of the target. Statistical learning also faces the following challenges: (1) complex and changeable pedestrian posture, clothing, scale and lighting environment. (2) The classifiers usually require large-scale training samples. (3) The quality of features directly affects the final detection performance of the classifier. Some progresses have been made in the use of multi-feature fusion and cascaded classifiers. Commonly used features include Haar feature [15], HOG feature [16], LBP feature [17], and Edgelet feature [18]. In this paper, to improve the efficiency of epidemic prevention, our goal is to compare and examine the procedures for identifying pedestrian and observing their social distance. Contributions of this work are:

- A novel vision-based surveillance system to monitor the social distancing violation among people, and at the same time, screening their body temperature at public areas.
- We applied a robust algorithm for people detection and distance measurement between the individuals. We propose faster and more accurate results in comparison to the other methodologies.
- The proposed technique is developed to an accurate approach of transforming a camera frame taken from a perspective point of view to top-down view. This will make the conversion rate between pixel distance and physical distance constant.
- The proposed approach could turn Thermal cameras in the present infrastructure capacity into smart cameras for social distancing and screening body temperature applications. This is to reduce the requirement for the number of hardware operations.

Recently, the deep learning-based methods have shown excellent performance in a series of computer vision-related tasks, including image classification [19], object detection [20], instance segmentation [21] and so on. Benefiting from the powerful non-linear fitting ability and data mining ability brought by massive parameters, it can usually extract high-level semantic features in images in a self-organizing manner without too much prior information. However, deep learning-based methods also have corresponding shortcomings when solving problems in practical applications. First, the large-scale parameters make the reasoning speed difficult to meet the real-time requirements in various complex scenarios. Second, deep learning models usually require a large number of samples to exert their performance. Finally, deep learning faces the optimization of many hyper-parameters, which puts forward higher requirements on the experience of researchers.

The motivation of this research is to minimize the spread of coronavirus among the individuals, and its economic impacts by providing AI-based solution. We propose a novel deep learning model (YOLOv4-tiny) in conjunction with implementation an algorithm for social distancing classification with bird’s-eye view to solve the issue of camera distortion.

After the introduction, the paper is organized as follows. Section 2 presents the related work and research background. Section 3 shows the overview of deep learning for object detection. Section 4 shows the experimental methodology of this research. Section 5 describes experiment results and discussion. Section 6 presents the implementation of the proposed techniques on the embedded system. Finally, conclusions and future work are drawn in Sect. 7.

## Related work

The combination of different features achieves the top performance on multiple datasets, including Caltech dataset and KITTI dataset. Zhang et al. [22] built a series of state-of-the-art performing pedestrian detectors by combining low-level features in the middle layer and enhanced decision forests. Kim et al. [23] applied the model compression technology based on the teacher–student framework to the random forest (RF) classifier to achieve fast and accurate pedestrian detection in low-spec surveillance systems. The experimental results show that the proposed method has better detection performance than several state-of-the-art methods in Performance Evaluation of Tracking and Surveillance 2006 dataset, Town Centre dataset, and the Caltech benchmark dataset.

To better extract global information, a multi-scale pedestrian detector based on self-attention mechanism and adaptive spatial feature fusion is proposed, and the spatial attention mechanism asymmetric pyramid non-local block (APNB) module is applied [24]. Nam et al. [25] illustrated that even with the emergence of complex and data-requiring methods, enhanced decision trees are still successful in the fast rigid object detection. Inspired by the recent work on the identification and decorrelation of HOG features, they proposed an effective feature transformation to remove the correlation in the local neighborhood, which is suitable for use with orthogonal decision trees. In fact, the orthogonal tree with local decorrelation features is better than the inclined tree trained on original features, and the computational cost is small. Magoo et al. [26] proposed a deep learning and surveillance video application framework based on bird’s-eye view, in which the YOLO v3 object detection model is utilized as a key point regression to detect key feature points.

Zhang et al. [27] proposed a pedestrian detector that combines common sense and daily knowledge into a simple and computationally efficient functional design. Experimental results on INRIA and Caltech pedestrian datasets show that their detector achieves the most advanced performance at low computational cost, and the proposed features are robust to occlusion. Walk et al. [28] how that the motion features derived from optical flow can significantly improve the image sequence, even in the case of low-quality video, it will also lead to flow field degradation. In addition, they also introduced a new feature, i.e., the self-similarity on the color channel, which can continuously improve the detection performance of static images and video sequences on different data sets. Finally, the authors discussed important complexity of detector evaluation and shows that the current benchmark protocol lacks key details, which may distort the evaluation.

By thoroughly analyzing and optimizing each step of the detection pipeline, Tome et al. [29] proposed a novel deep learning architecture better than the traditional method, which achieved the task accuracy close to the most advanced method and requires low computing time. Finally, the author tested the proposed method on the edge computing suite NVIDIA Jetson TK1, which serves as a 192-core platform that is conceived as the leading computing brain for autonomous vehicles of the future. To overcome the low-resolution and low-signal-to-noise characteristics of infrared images that may vary depending on the weather, Chen et al. [30] proposed a novel attention-guided encoder–decoder convolutional neural network. In addition, they also proposed an attention module to re-weight the multi-scale features generated by the encoder–decoder module. On KMU and CVC-09 pedestrian data sets, the proposed method improves the accuracy of the most advanced methods by 5.1% and 23.78%, respectively.

Lin et al. [31] proposed a texture aware-based deep feature learning method for pedestrian detection, which combines the fine-grained information into hidden convolutional features to make them more distinguishable from human parts. Comprehensive experimental results on four challenging pedestrian benchmark datasets demonstrated the effectiveness of the proposed method. Li et al. [32] proposed two novel deep learning methods. The depth direction separable convolution and linear bottleneck techniques are used to reduce the computational cost and the number of parameters. Six strategies are used to enhance the pedestrian images collected on foggy days to enrich the database. Experimental results show that the proposed method can effectively detect pedestrians in hazy weather and is significantly better than the existing methods in accuracy and speed.

Cao et al. [33] proposed a unified framework called multi-layer channel feature (MCF) to overcome the disadvantages of ignoring features. It integrates each layer of HOG + LUV and CNN into a multi-layer image channel. The weak classifier in each level of the multi-level cascade is learned from the image channel of the corresponding layer. Experiments were carried out on Caltech dataset, INRIA dataset, ETH dataset, TUD Brussels dataset, and KITTI dataset. With richer features, MCF has reached the most advanced level in the Caltech pedestrian dataset (i.e., 10.40% failure rate). Li et al. [34] developed a model that introduces multiple built-in subnetworks that use the scale of disjoint ranges to detect pedestrians. The outputs of all subnets are adaptively combined to generate the final detection results, which is robust to large changes in the size of the instance. Extensive evaluation on several challenging pedestrian detection data sets well proved the effectiveness of the method.

To estimate social distance violations between people in [35], the authors used an approximation of the physical distance of pixels and set a threshold. The violation threshold is established to evaluate whether the distance value violates the minimum social distance threshold. The research results showed that with YOLOv3, the developed framework successfully distinguishes individuals who walk too close and who violate social distance. Ahamad et al. [36] calculated the distance between people detected in the captured shot and compared it with a fixed pixel value. In the segmented tracking area, the distance between the center point and the overlapping boundary between the person is measured. By detecting the unsafe distance between people, an alarm or warning can be issued to keep the distance safe. Taking the video frame of the camera as the input, Hou et al. [37] used the deep learning methods to evaluate the distance between people. The video frame is converted into a top-down view for distance measurement from the 2D plane.

Rezaei et al. [38] developed a hybrid computer vision and deep neural network (DNN) model based on YOLOv4 to monitor social distance to prevent the spread of COVID-19. This may help authorities redesign the layout of public places or take preventive measures to mitigate high-risk areas. Ksentini et al. [39] recommended combining the Internet of Things and Multi-Access Edge Computing (MEC) technology to build a service that can check and warn people who are not socially distancing in near real time. The proposed service consists of a client application installed on the user’s smartphone, which periodically sends GPS coordinates to a remote server located at the edge of the network (i.e., MEC). Yang et al. [40] proposed an active monitoring system to slow down the spread of COVID-19 by warning individuals in the region of interest. First, we introduce a vision-based real-time system, which can detect SD violations and send non-invasive audio-visual prompts using the most advanced deep learning model. Second, we define a new critical social density value and show that if the pedestrian density remains below this value, the chance of SD violation can remain close to zero.

## Deep learning for object detection

Deep learning method is the object detection approach, which mitigates the computational complexity issues by emulating the tasks for predicting the objects in the images. Convolutional neural network (CNNs) is a type of neural network that is efficient for patterns capturing in the multidimensional spaces. Convolutional neural network is widely used in deep learning architectures for object detection. This algorithm takes an input image and assigns biases and the learnable weights for different classes in the images and differentiates them from one class to another. There are various types of CNN models that are applied in different applications for object detection. In [41], Ross Kirchick proposed a regional convolutional neural network detector. R-CNN consists of four different stages. The algorithm first initiates an image into the input layer and takes out the targeted features from the image, after that it computes the extracted features throughout different convolutional neural network layers, and lastly it classifies the extracted features. R-CNN uses selective search technique to produce region proposals from the image. This architecture takes a large amount of the computational time to classify the regions for each image. Regional convolutional neural network cannot be applied in real-time application for object detection. R-CNN is multi-stage pipelines. It is slow because it executes a ConvNet forward pass for each regional proposal, without sharing computation. R-CNN extracts the features from each object proposal in each test image. It takes a long time for predicting the objects in each image. Object detection with R-CNN takes 47 s/image (on GPU), which makes it slow for real-time applications in addition to that, regional convolutional neural network cannot be trained at one time, instead R-CNN is required to train every part in the image separately.

Fast-Regional convolutional neural network is another approach for object detection [42]. Fast R-CNN improved the drawback of regional convolutional neural network architecture and creates a faster model for object detection. Fast-RCNN has the same structure of R-CNN detector, in which the input image is introduced into convolutional neural network to produce convolution feature maps, rather than providing regional proposals to layers of CNN. The region proposal is warped into small squares in this architecture. Region of interest (ROI) uses pooling layers, and these regions are redesigned into a fixed size that can be provided into the fully connected layer. Softmax layer is utilized in this model to predict the objects and the offset costs of bounding box.

Fast R-CNN is also using selective search method to predict the region proposals. This technique is slow and time-consuming which is influencing the neural network model performance for real-time application.

Recent techniques have been proposed for object detection include deep learning models such as YOLO detectors. You only look once or (YOLO) is a state-of-art object detector, which is targeted for real-time applications. YOLO was proposed by Joseph Redmon et al. in 2016 [43]. This algorithm uses a single neural network for the entire image. YOLO splits the images into small regions and generates the bounding boxes and the probabilities of the classes for each region in the image. The bounding boxes are weighted by predicted probabilities.

**Fig. 2:**
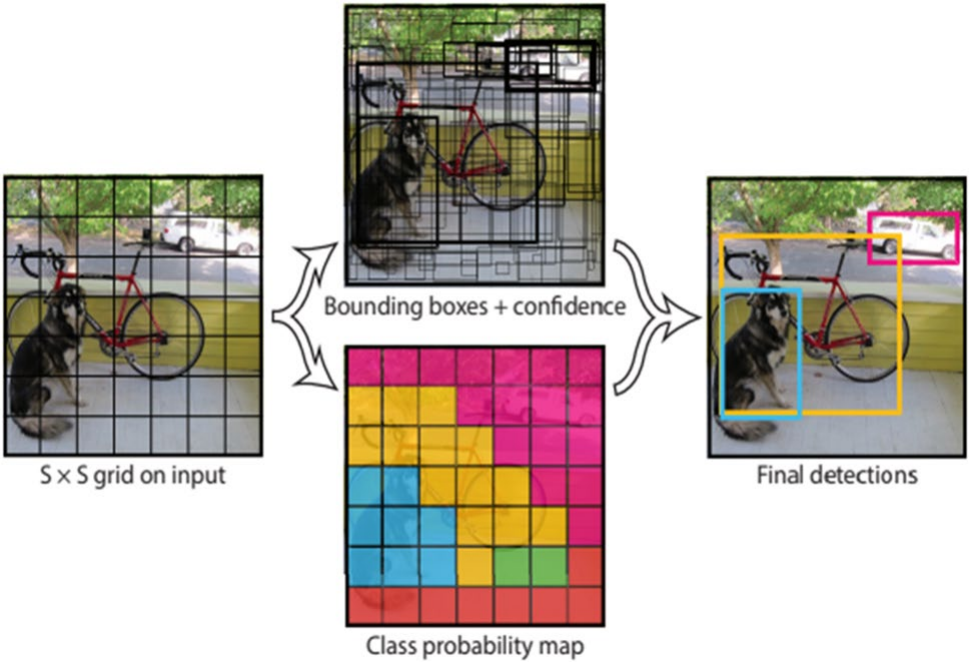
YOLO for object detection: input image which divides into S × S grids, each cell predicts the bounding boxes and the confidence scores, and lastly, the score generates the probability of the detected class with the enclosed bounding box. Adapted from [43]

YOLO detector looks the whole image at once; therefore, its detection is obtained by the whole information in the image. YOLO uses single network evaluation, which unlike the regional convolutional neural network detectors (R-CNN and Fast-RCNN) which need thousands of regions for one image. The model obtains the image input features and splits it into *S* ×  *S* grid. After that, it extracts the data from each grid in the introduced image and uses the confidence score of the detected object to produce the rectangle box, as seen in Fig. 2. Each cell predicts the bounding box and confidence score. The bounding box contains five prediction parameters, which are determined by (*x*, *y*, *w*, *h*) and the confidence score value, where (*x*, *y*) coordinates represent the center of the bounding box, and (*h*, *w*) reflects the height and the width of the whole entire image. The confidence scores represent the measurement of how confident the architecture is that the box contains of the object to be predicted. YOLO has several improvements and produces series of the deep learning architectures. The first three yolo versions (YOLO, YOLOv2 [44], and YOLOv3 [45] were released in 2016, 2017, and 2018, respectively, by Joseph Redmon. However, YOLOv4 has been explored as an optimal speed and accurate object detector. This model has significant improvements to the previous versions. YOLOv4 extracts the influence of state-of-art bag of freebies (BoF) and several bag of specials (BoS). The bag of freebies enhances the accuracy of the detector, without increasing the influence time. They increase the training cost. In contrast, the bag of specials increases the influence cost by a small amount. However, they enhance the accuracy of object detection significantly. YOLOv4 [46] is also based on Darknet and has achieved an AP of 43.5% on COCO dataset with real time of 65 *fps* on Tesla 100. YOLOv4 is considered the fastest and the most accurate model in terms of both accuracy and speed. Moreover, YOLOv4-Tiny [47] is a light-weight version of YOLOv4 architecture. This model is simple to construct, and it has satisfactory performance in object detection. This algorithm can minimize the computational complexity on assumption for ensuring the accuracy of the neural network model.

## Methodology

### The architecture of social distancing

**Fig. 3:**
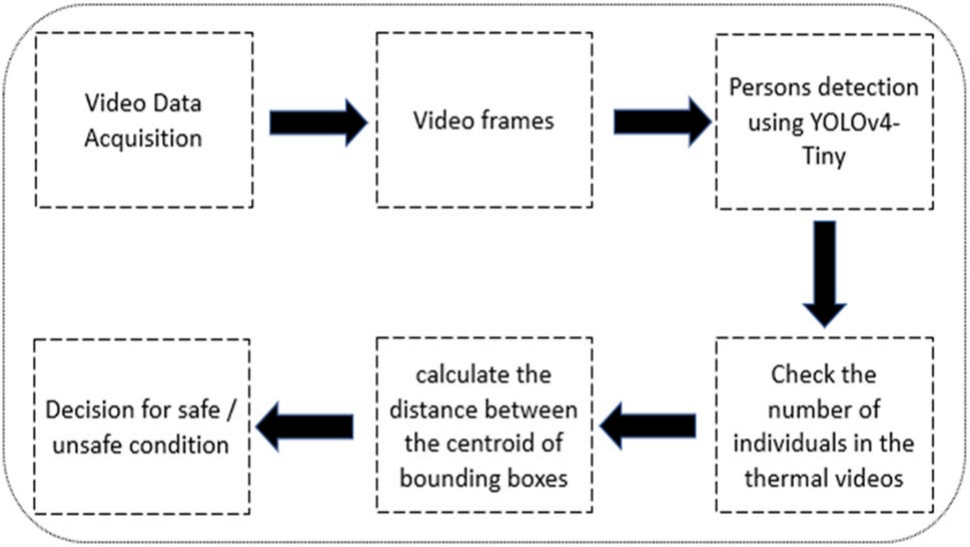
Sequence design for social distancing architecture

In this section, we will discuss the essential steps that are required to build a sequence design to determine and check if the social distancing rules are respected or not among the individuals on the thermal videos as seen in Fig. 3:

1. Streaming the thermal videos, which contains the individuals.
2. Extracting the thermal video into frames.
3. Applying YOLOv4-Tiny architecture to detect only the individuals in thermal videos.
4. Verify the number of the individuals that are in the thermal videos.
5. Calculate the distance between the center point of the bounding boxes that contains the individuals in the thermal videos.
6. Lastly, the algorithm will make the decision for violation or safe conditions for the individuals based on the number of individuals in the thermal videos, and the measured distance between the centroid of bounding boxes. This is to note that we made two different levels for violation with two different threshold set points for the measured distance between the center points of the bounding boxes. First violation level is called Alert, which is marked with a yellow color for the bounding box, and the second violation level is defined as Risk, which is marked with a red color for the bounding box. We marked the safe condition with a green color for the bounding box.

**Fig. 4:**
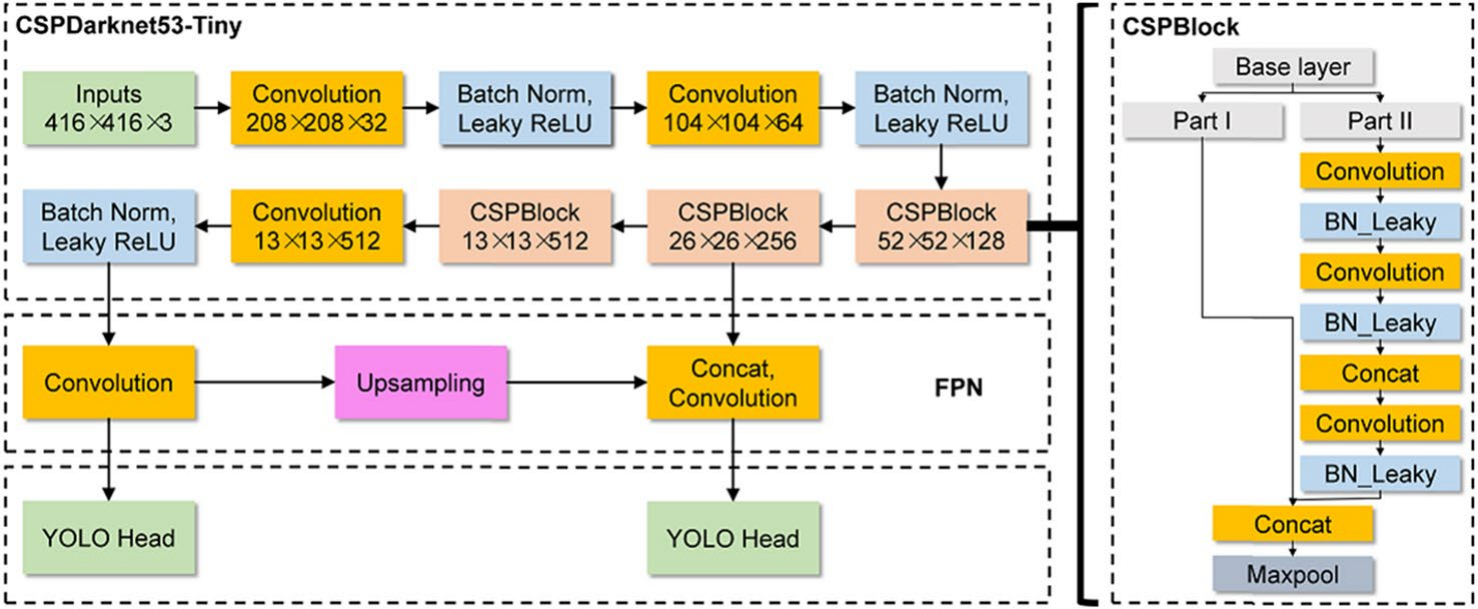
The architecture of YOLOv4-tiny Network

### Object detection

Object detection is a computer vision approach, which locates the objects in an image or video. Identifying the coordinates of the individuals in the thermal videos is the first stage of this research. We used YOLOv4-tiny for people detection in the thermal videos. We built the CNN with 37 layers for YOLOv4-tiny. This research aims are to design a light-weight model to account the requirement of CNNs’’ real-time application in low-cost embedded systems such as IoT devices. The proposed approach is compliant to YOLOv4 model. The total weight is reduced for the final deep learning model. YOLOv4-Tiny utilizes a couple of different changes from the original YOLOv4 network to achieve fast execution on low-cost embedded system. First and foremost, the number of convolutional layers in the CSP backbone are compressed. In addition, the number of YOLO layers has been reduced to two instead of three and there with few anchor boxes for prediction. YOLOv4-tiny consists of three major modules as shown in Fig. 4, i.e. CSPDarknet53-Tiny, Feature Pyramid Network (FPN), and YOLO Head. CSPDarknet53-Tiny is utilized for primary feature extraction, which is formed of Convolutional block (Conv) and CSPBlock. Convolutional layers include batch normalization and activation functions. Batch normalization is used to regulate the model. It substituted the requirement of using the dropout layers in the architecture to eliminate the overfitting problems. It enhances the normalization for its input by defining the variance values. The activation functions are leaky ReLu (Rectified Linear Unit). According to the systematic characteristics of Cross Stage Partial Network (CSPNet), CSPBlock splits the model of the Base layer into two parts. The first part is created as residual edge, and the other part is composed with the first one to produce the final output after a series of convolutional operations. FPN structure can merge the features of various network layers, which can reserve semantic data of deep network and geometric information of low-level network. Thus, this is to enhance the ability of feature extraction. YOLO Head is the final module of the architecture for feature output results. The role of YOLO Head in the case of a one-stage detector to carry out the dense prediction. The dense prediction is the final result, which is composed of a vector containing the coordinated of the predicted bounding boxes (height, width, center), the label, and the confidence score of the prediction, see Eq. (1), where $${P}_{\mathrm{w}}$$ and $${P}_{\mathrm{h}}$$ represent the width and height of the bounding boxes. ($$Cx,Cy)$$ represents the coordination of the top left corner of the image:

$$bx=\sigma \left({t}_{x}\right)+ Cx,$$

1 $$bx=\sigma \left({t}_{y}\right)+ Cy,$$

$${b}_{\mathrm{w}}= {P}_{\mathrm{w}} \cdot {e}^{tw},$$

$${b}_{\mathrm{h}}= {P}_{\mathrm{h}} . {e}^{th}.$$

### Euclidean distance and violation thresholds

At this stage of this work, after individuals are detected in the thermal videos, the Euclidean formula is used to measure the distance between each detected centroid pair using the enclosed bounding boxes and their centroid information; see Eq. (2). The calculation distance is computed with the scaling factor to obtain real-world metrics. The distance for the Euclidean measurement is defined as 6 feet (approximately 180 cm). Two different predefined minimum social distance violation rules are determined to distance assumptions. Considering that, we set two different thresholds for these violations as yellow and red colors for the detected bounding boxes. The violation of the first threshold is defined as “Alert”, which is marked with a yellow color while the second assumption of violation is defined as “Risk”, which is marked with a red color. The bounding box’s color is formerly initiated as green, if the distance is >6 feet between the detected bounding boxes. If the distance is ≤6 feet, and >5 feet (the first threshold value “Alert”), the bounding boxes color are updated to yellow, and if the distance is ≤5 feet (the second threshold value “Risk”) between the detected individuals, the bounding boxes color are changed to red, which means the social distancing is not maintained:

2 $$d(C1, C2)\hspace{0.17em}=\hspace{0.17em}\sqrt{{{(x\mathrm{max }- x\mathrm{min})}^{2}+ (y\mathrm{max }- y\mathrm{min})}^{2},}$$

where *d* is the distance between the centroid of bounding box.

### Bird’s-eye view transformation

**Fig. 5:**
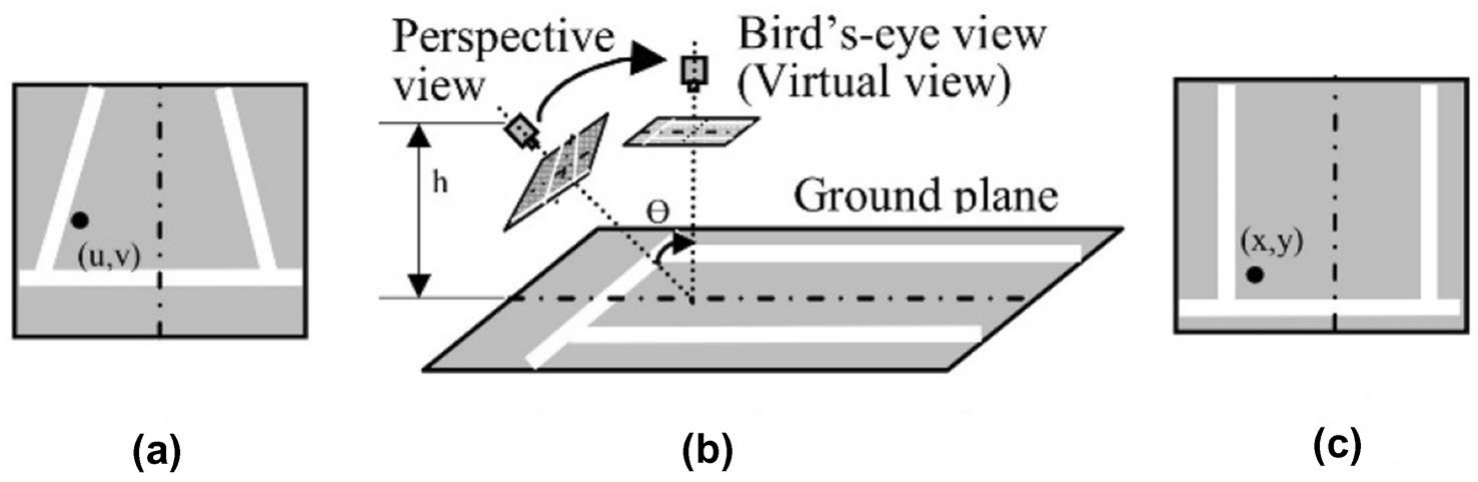
Perspective transformation to bird’-eye view: **a** perspective view image.**b** Perspective transformation.** c** Bird’’s-eye view image

Based on the previous work that we carried out for people detection and social distancing measurement on thermal images [48], it was discovered that the bounding boxes identifying nearby persons were larger, and the bounding boxes identifying the distant were not accurate due to the perspective effect. This perspective causes a distortion, and the distance between the individuals cannot be computed by calculating the pixels directly. Therefore, in this research, we decided to implement the proposed approach by converting the perspective view into bird’s-eye view, and in such way, the distances between the individuals can be identified by the pixel/meter scale measurement. The novelty of our method is that we implemented the top-view computer vision to enhance the scalability of the system for monitoring the individuals as the camera will not be required to be configured in a specific manner. To make the bird’s-eye view transformation, it is essential to calculate the matrixes, which are required for bird’s-eye view transformation. The transformation is referred to reverse perspective mapping (RPM). RPM takes as input frontal view, and then it applies the homography, and establishes a top-down view of the captured scene. Figure 5 shows the captured image from a perspective to top-down view (bird’’s-eye view), where the dimensions in the image have a real interconnection with real-world dimensions [49]. The transformation matrix (TM) is computed using a function in OpenCV Library, which is defined as “*get Perspective Transform*”; see Eqs. (3) and (4). The calculation is performed by expecting the person utilizing the prototype will identify the source and the target points. On that account, the tasks of predicting the coordinate points of the detected persons on source of images were specified to the user who will perform the prototype. In addition to that, the bird’s-eye view matrix is achieved by multiplying each element matrix from the source of images using function “*warpperspective*” in OpenCV Library; see Eq. (5):

3 $$\left[ {\begin{array}{*{20}c} {t_{i} {x_{i}^{\prime}} } \\ {t_{i} {y_{i}^{\prime}} } \\ {t_{i} } \\ \end{array} } \right]^{ } = {\text{TM}}* \left[ {\begin{array}{*{20}c} {x_{i}^{ } } \\ {y_{i}^{ } } \\ 1 \\ \end{array} } \right],$$

where TM is the transformation matrix.

4 $$dst(\acute{\iota}) = \left( {{x_{i}^{\prime}} , {y_{i}^{\prime}} } \right), \quad src \left( i \right) = \left( { x_{i} , y_{i} } \right), i$$

5 $$St \left(x,y\right)=src \left(\frac{T{M}_{11}x,T{M}_{12}y,T{M}_{13}}{T{M}_{31}x,T{M}_{32}y,T{M}_{33}},\frac{T{M}_{21}x,T{M}_{22}y,T{M}_{23}}{T{M}_{31}x,T{M}_{32}y,T{M}_{33}}\right),$$

where *dst* defines the target matrix achieved after the transformation, and *src* is the source matrix, which is transformed. It is calculated to convert each element of the source matrix to each element of targeted matrix.

### Dataset training procedure

**Fig. 6:**
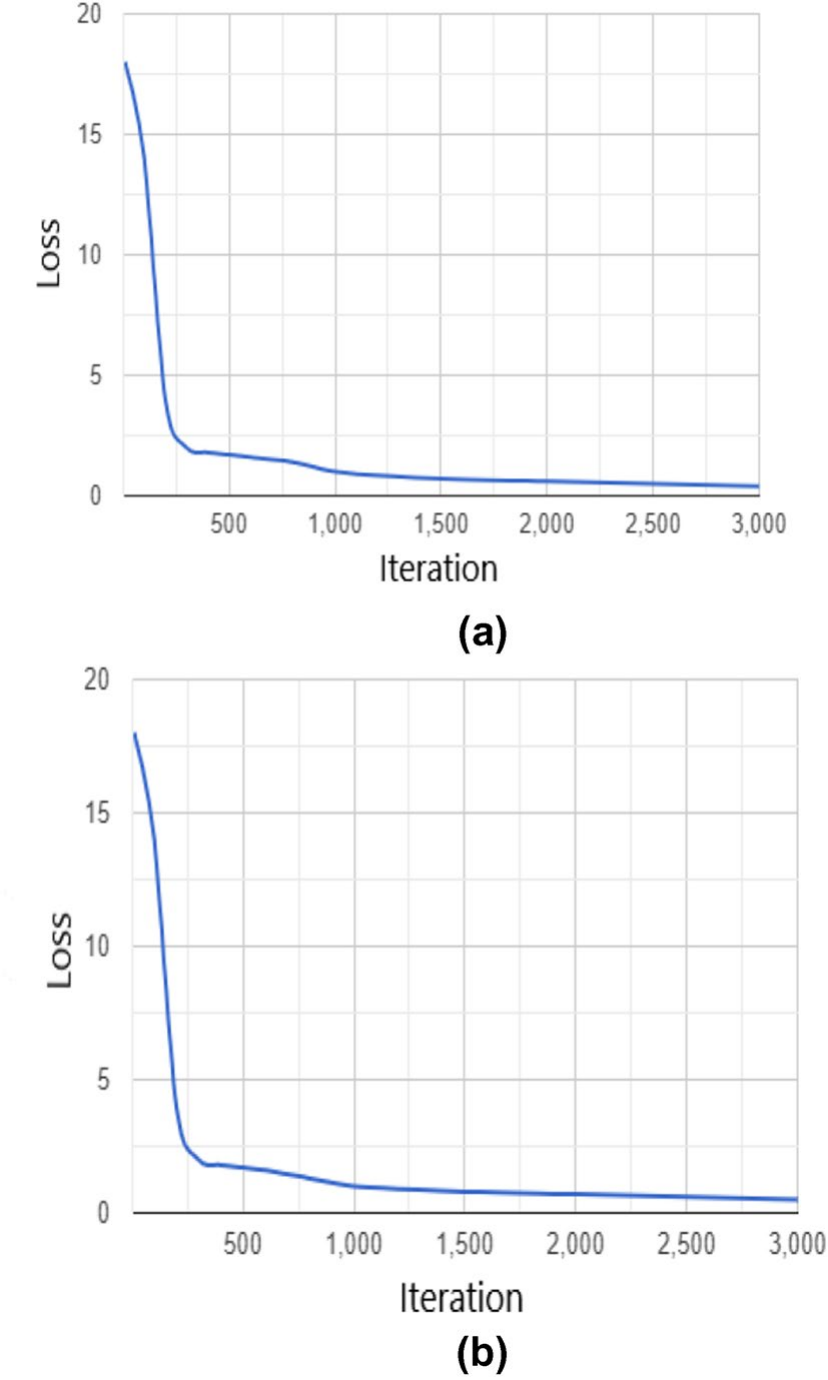
**a** Training loss curve for dataset I.** b** Training loss curve for dataset II

The proposed approach was trained with two different datasets of thermal images. Dataset I consist of 1000 images. These images were collected by FLIR for thermal cameras [50]. This dataset features an initial set of images from thermal cameras, which utilized infrared radiation sensors. Dataset II contains 950 images of different people, which were taken from realistic condition that were happened during surveillance and monitoring area. They are taken from different scenes, including people sneaking, walking, running, and people in different body positions. These images were collected from different resources on the internet. The images of both datasets were labeled for class of only persons in the images. The images have been split into 70% for training, 20% for validating, and 10% for testing the architecture for each dataset. YOLOv4-tiny has been trained with stochastic gradient descent (sdgm) [51]. The learning rate has been optimized in the training option to control the model response to the error. The learning rate value was fine-tuned at 10^−3^, and the loose curve was steady with this value for both datasets, see Fig. 6.

Table 1 shows the training hyper-parameters for YOLOv4-tiny architecture. For all our experiments in this research, we used NVIDIA Tesla K80 GPU training system on Google colab cloud service, which has been designed to train neural network architectures for object detection and image classification tasks.

**Table 1:** Training hyper-parameters for the proposed neural network

Parameter	Method
Training options	Sdgm
L2 regularization	0.05
Number of iteration	3000
Mini-batch size	16
Learning rate	0.001

## Experiment results and discussion

In this section, all experiments details and comparison are described. We illustrated the experiments from different perspectives. To evaluate the performance of the proposed approach, we run the algorithm over the testing thermal images of both datasets. The thermal images were composed from realistic situation by different thermal cameras in the indoor and outdoor environments. Thermal cameras can perform the measurement for radiated emitted energy from the skin of human in a safe and fast manner. With this in mind, we chose these datasets for our experiments. We also applied the YOLOv4-tiny and the technique proposed for social distancing measurement with bird’s-eye view on large scale of thermal videos. These videos are scalable of screening individuals’ movement while these thermal cameras were measuring their skin temperature. Further to our exploration, we carried out other experiment by examining (Fast R-CNN) and you only look once (YOLOv2) detectors for people detection, using the same thermal images of the two training datasets of thermal images. This is to compare these architectures with YOLOv4-tiny and proposed techniques in Sect. 4 using the same testing thermal images from both datasets and thermal videos database. For metric computation, confusion matrix criteria have been used to assess the proposed algorithm. The metrics that have been chosen to analyze the goodness for the algorithm are recall, accuracy, and precision; see Eq. (6):

$${\text{Precision}} = \frac{{{\text{TP}}}}{{{\text{TP}} + {\text{FP}}}},$$

6 $${\text{Accuracy}}\, = \,\frac{{{\text{TP}} + {\text{TN}}}}{{{\text{TP}} + {\text{FN}} + {\text{TN}} + {\text{FP}}}},$$

$$\,{\text{Recall}} = \,\frac{{{\text{TP}}}}{{{\text{TP}} + {\text{FN}}}},$$

where TP represents the number of true positive; TN represents the number of true negative; FP represents the number of false positive; FN represents the number of false negative.

**Fig. 7:**
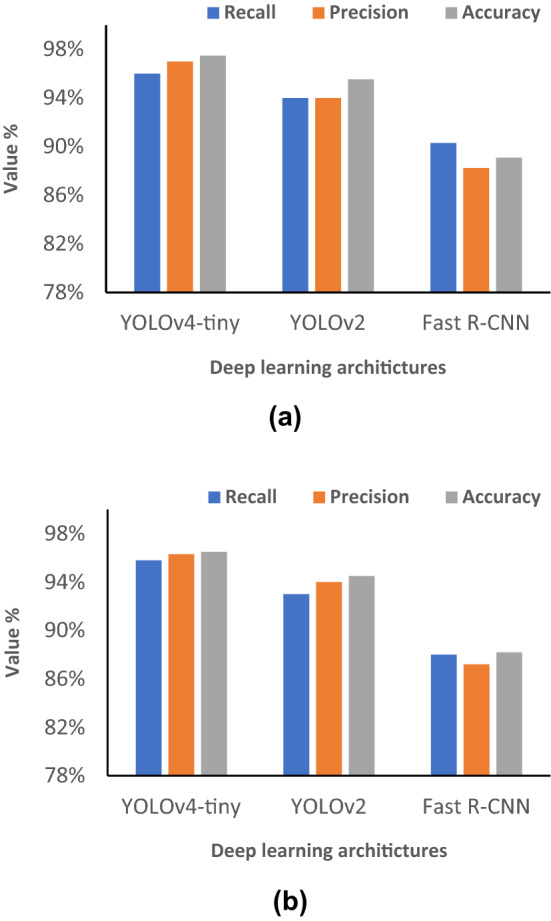
**a** Performance of YOLOv4-tiny vs. other object detectors with Dataset I.** b** Performance of YOLOv4-tiny vs. other object detectors with Dataset II

**Fig. 8:**
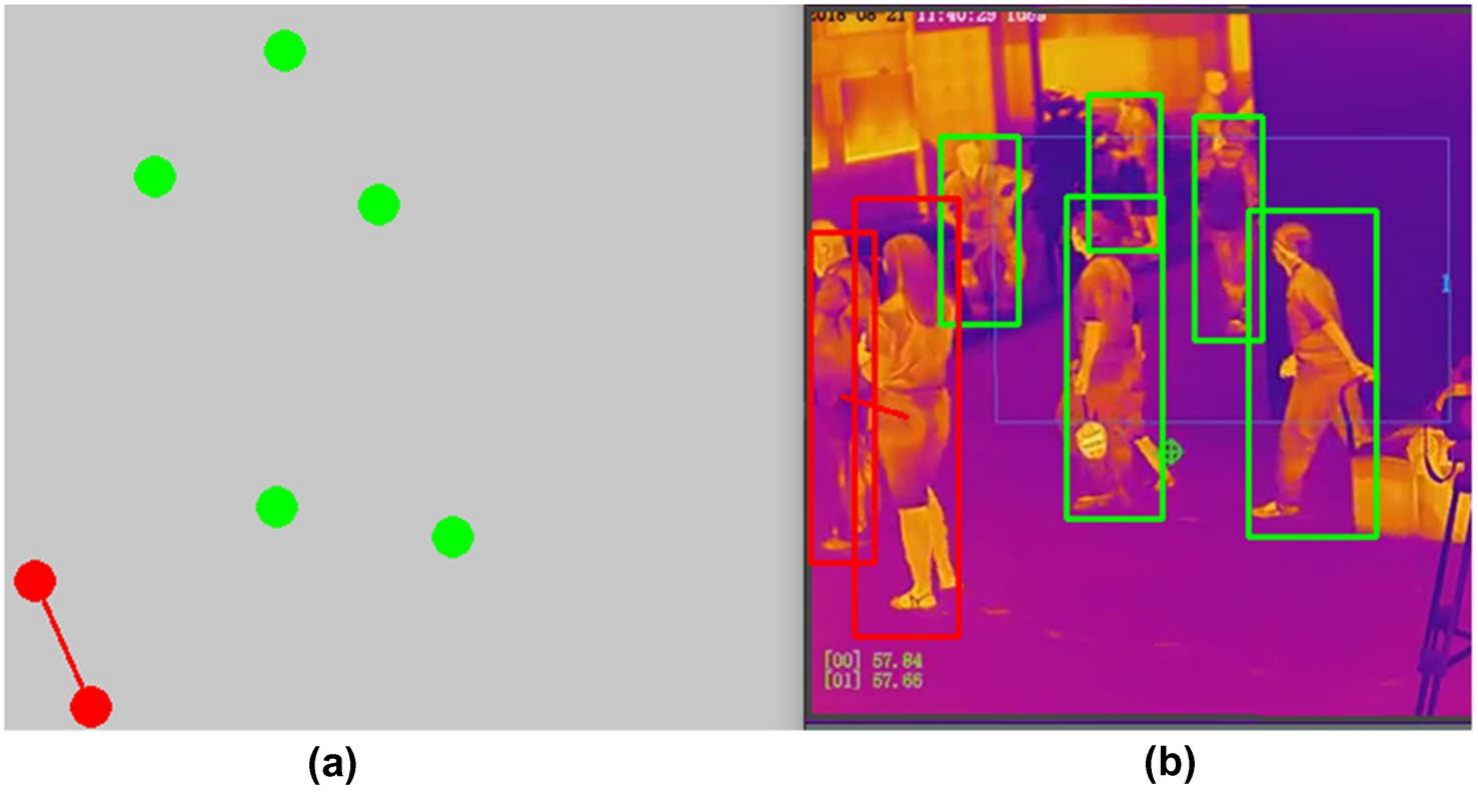
Social distancing status with the proposed method, which show two persons violated the Risk threshold distance, and five persons were in safe conditions:** a** perspective transformation of human detection points with bird’s-eye view.** b** People detection with enclosed bounding boxes in the thermal videos

Based on the results from these experiments, YOLOv4-tiny achieved promising results for people detection on the thermal images on both testing datasets and thermal videos database; see Fig. 8. It showed better performance and overcame Fast R-CNN and YOLOv2 architecture; see Fig. 7. The birds’ eye view has been displayed also in separate window as shown in Fig. 8. Points for human detection have been shown in bird’s-eye view window for both safe and risk conditions with assigned colors, respectively. Furthermore, YOLOv4 showed improvement in terms of accuracy vs other methodologies [52–54]; see Table 2.

**Table 2:** Performance of the proposed approach vs. other methodologies

Method	Accuracy (%)
YOLOv4-tiny with Dataset I	97.48
YOLOv4-tiny with Dataset II	96.5
Sener et al. [52]	93.3
Rinkal et al. [53]	92.8
Yadav et at [54]	91

It can be seen that the detection performance has been improved using YOLOv4-tiny verses the other two architectures. YOLOv4-tiny model uses a modified path aggregation network, and a modified spatial partial pyramid pooling, which all are utilized to enhance the accuracy of the architecture for object detection. In addition to that, YOLOv4-tiny uses CSPDarknet53 as backbone neural network in the model. CSPDarknet53 added Cross Stage Partial Network (CSPNet) on every large residual block of Darknet53 and integrated into mapping the features from the images. This feature map is divided into convolution operation and the other with a combination of the last convolution result. Therefore, CSPDarknet53 can effectively increase the learning ability of CNNs’ layers and minimize the computation cost, which enables the network to obtain more accurate detection capability.

## Implementation on the embedded systems

### GPU-based systems

In recent years, manufacturers have been forced to find alternatives to the traditional source of computational power increase. Due to the fundamental limitation in the fabrication of integrated circuits, it is no longer feasible to rely on upward processor clock speeds as a means of extracting additional power from existing architecture. The release of Graphic Processor Units (GPUs) that possessed pipeline attracted many researchers to the possibility of using graphics hardware for many applications. With this in mind, NVIDIA released GPUs for professional applications in the market. NVIDIA expanded in many applications including the acceleration of AI and deep learning architectures. In addition to that, NVDIA provides an application programming interface, which is known as CUDA or Compute unified device architecture. It allows the creation of parallel computation, which utilizes GPUs. NVDIA released various developer kits such as Jetson nano, Jetson TX1, Jetson TX2, and Jetson AGX Xavier for edge computing. These devices are incredible for AI performance, which are targeted for real-time applications. In our research, we will utilize Jetson nano and Jetson AGX Xavier for execution of the proposed technique.

### Social distancing on embedded systems

To assess the performance for the proposed techniques, specific tests have been carried out to examine the dependency of the proposed technique computational cost on the targeted hardware. We deployed the algorithm as standalone applications in two different NVIDIA devices: Jetson nano and Jetson AGX Xavier. V2 raspberry PI camera has been used and exposed it to another computer device, which displayed set of videos, which were obtained from thermal cameras. We performed the test measuring the maximum frames per seconds *fps* that can be obtained and we compared the achieved results with other methodologies. Results are reported in Table 3. Based on the results from our experiments, NVIDIA Jetson Xavier achieved the best results for frames per seconds, which is reaching 23 fps while the real time for NVIDIA Jetson nano has reached only 11 fps. In such configuration, the Xavier board is 2× times faster than the NVIDIA Jetson nano.

**Table 3:** The real-time measurement for proposed approach vs other methods

Method	Real time in (fps)
This work on Xavier device	23
Ahmed et al. [55]	20
This work on Jetson nano	11
Bilal et al. [56]	11
Pouw et al. [57]	10

Table 4 reports the power consumption of Jetson nano and Jetson AVG Xavier using different configurations. The NVIDIA devices were disconnected from any peripherals, such as the monitor, mouse, and keyboard. While during the execution of the algorithm, the power consumption of Jetson nano was recorded at 3.21 W, while the power consumption of Jetson Xavier was measured 17 W.

**Table 4:** Power consumption measurement in different scenarios

NVIDIA devices status	Power measurement (W)
Jetson nano without accessories	3.21
Jetson nano with accessories	5.5
Jetson Xavier without accessories	17
Jetson Xavier with accessories	18.3

GPU (graphics processing unit) and CPU (central processing unit) performance are critical parameters of algorithm success in the low-cost IoT devices. With an efficient CPU and GPU, this will help the execution the neural network model with less heat and noise in the hardware, Graphic processing unit works together with central processing unit in the NVIDIA system to enhance the throughput of data and number of comprehensive computations within the application. The GPU and CPU performance are important parameters for the NVIDIA system to assess the capability of AI algorithm execution. Table 5 illustrates the CPU and GPU performance and temperature measurement while the proposed technique is running on NVIDIA (Jetson Xavier and Jetson nano) devices.

**Table 5:** Temperature measurement and % utilization for the GPU and CPU in Jetson Nano and Jetson Xavier while our method is running on them

	Performance (%)	Temperature (°C)
Jetson nano (GPU)	98	57
Jetson nano (CPU)	72.1	53.1
Jetson Xavier (GPU)	86	62
Jetson Xavier (CPU)	50	57

**Fig. 9:**
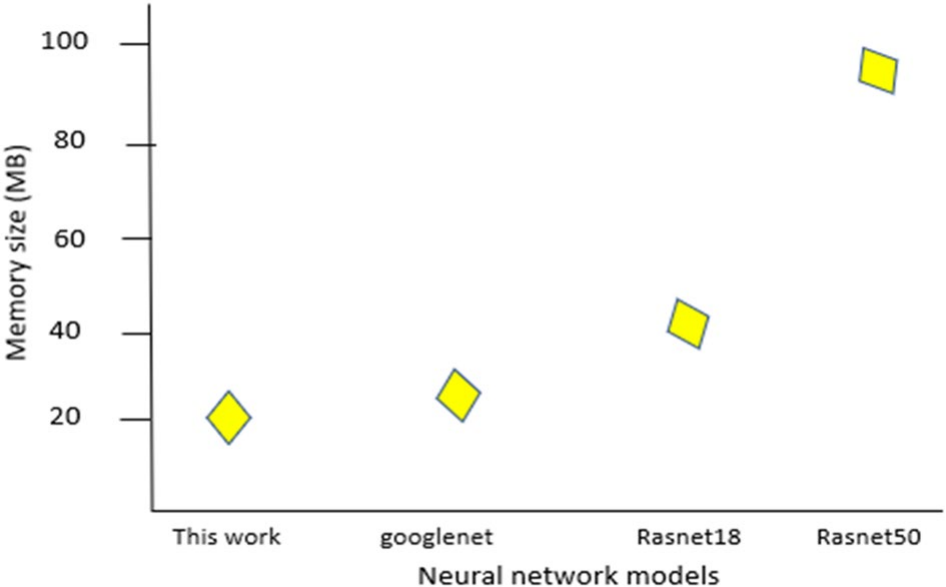
Comparison of the proposed technique verses other pretrained neural network models in terms of memory size

This research aims to draw a comparison between the proposed method verses the other available state-of-art pretrained neural network architectures. The benefit of the proposed technique is its light-weight memory size on disk (~21 MB) and consequently with small number of trainable parameters, which makes it suitable for low-cost IoT devices. Other methods use massive CNN layers which translate larger memory size on disk such as Rasnet50 model in method [43]. In addition to that, these pretrained architectures will have low performance for real-time detection while executing on low-cost IoT devices, and this will affect human visual recognition for tracing the objects. Figure 9 shows the memory size on disk for YOLOv4-tiny and other pretrained neural network models.

### Further discussion

This research is an implementation from our recent work in method [48]. In this work, we used novel deep learning model YOLOv4-tiny, which is a predecessor to the utilized model YOLOv2 in the previous work. YOLOv4 is newly released, which is accurate for object detection that can be trained with smaller batch size on single GPU. The performance of YOLOv4-tiny showed better results verses YOLOv2 for object detection. Although the memory size on disk was less in the previous research which was only 14 MB in comparison to measured memory size in this work 21 MB. This is due to the number of neural network layers in YOLOv4-tiny is more than the previous approach with YOLOv2 model, which is indeed enhanced the accuracy for people detection. YOLOv4-tiny model has also been tried to enhance the performance in comparison to YOLOv2, which has been utilized in the previous work. The input adopts a number of advanced algorithms in the field of deep learning, such as CSPDarknet53 backbone. In this work, we improved the violation levels versus the previous research by determining three different threshold rules to maintain social distancing. We set three different level of assumptions, which define safe, alert, and risk that are marked with green, yellow, and red, respectively. This is to analyze and formulate the transmission of COVID-19 and evaluate the control strategies for the pandemic by the authorities. The proposed technique has been examined in two different NVIDIA devices in (Jetson nano and Jetson Xavier). This is to have further exploration for the computational cost in different embedded devices for the executed algorithm such as power consumption, real-time detection, and CPU and GPU utilization use. In addition to that, we improved the issue with the perspective effect that was encountered in the previous research. It has been implemented to transform the perspective view into the bird’s-eye view. In this way, the distance between the detected individuals can be visualized clearly. Once we have the coordinates of the individuals in the top-down view, the social distancing parameters become straightforward. Table 6 illustrates the difference for the obtained parameters between the proposed approach in this research and the previous work in [48].

**Table 6:** The difference for the obtained parameters between the proposed approach in this research and the previous work in method [48]

	This work	Method (48)
Accuracy	97.48%	95.6%
Memory size on disk	21 MB	14 MB
Number of neural layers	37	29

## Conclusion and future work

This research proposed a deep learning-based social distancing technique using bird’s-eye view for people detection on thermal videos. The achieved results confirmed that the developed intelligent surveillance system identified the individuals who violated the social distancing and at the same time screening their body temperatures. YOLOv4-tiny showed good performance in terms of accuracy and precision in comparison to the other deep learning models. Bird’’s-eye view technique has been developed for mapping the human detection points effectively. The proposed approach is a solution for the authority to visualize the pedestrians if they comply with rules for social distancing in indoor and outdoor areas. We defined the safe condition with green color for the bounding boxes while unsafe condition has been determined with two different assumptions of violation: alert and risk, which have been marked with yellow and red colors, respectively. The implemented algorithm has been successfully deployed in the NVIDIA systems (Jetson nano and Jetson AGX Xavier) and showed real time for person detection for up to 23 frames per seconds which is suitable for human visual recognition. In the future, we will implement the proposed approach using novel deep learning detector (YOLOv5) for people detection. Moreover, the deployment for the proposed technique on FPGA will be considered.
